# A New Protocol to Detect Multiple Foodborne Pathogens with PCR Dipstick DNA Chromatography after a Six-Hour Enrichment Culture in a Broad-Range Food Pathogen Enrichment Broth

**DOI:** 10.1155/2013/295050

**Published:** 2013-12-02

**Authors:** Masahiro Hayashi, Tatsuya Natori, Sayoko Kubota-Hayashi, Machiko Miyata, Kiyofumi Ohkusu, Keiko Kawamoto, Hisao Kurazono, Souichi Makino, Takayuki Ezaki

**Affiliations:** ^1^Division of Anaerobe Research, Life Science Research Center, Gifu University, 1-1 Yanagido, Gifu 501-1194, Japan; ^2^Department of Microbiology, Gifu University Graduate School of Medicine, 1-1 Yanagido, Gifu 501-1194, Japan; ^3^Division of Food Hygiene, Department of Animal and Food Hygiene, Obihiro University of Agriculture & Veterinary Medicine Inada-cho, Obihiro, Hokkaido 080-8555, Japan; ^4^Department of Domestic Science, Kyoto Seibo College, Kyoto 612-0878, Japan

## Abstract

A quick foodborne pathogen screening method after six-hour enrichment culture with a broad-range food pathogen enrichment broth is described. Pathogenic factors of *Salmonella enterica*, *Shigella* spp., enteroinvasive *Escherichia coli*, and enterohemorrhagic *E. coli* are amplified with a cocktail primer and rapid polymerase chain reaction (PCR), which finishes amplification in 30 min. The PCR amplicon was differentiated with a dipstick DNA chromatography assay in 5–10 min. Starting from a four- to six-hour enrichment culture, this assay was finished within 45 min. Detection sensitivity of this protocol was less than 2.5 CFU/25 g for *S. enterica* and 3.3 CFU/25 g for enterohemorrhagic *E. coli* in spiked ground meat experiments.

## 1. Introduction

Infectious gastroenteritis is a leading cause of morbidity and mortality worldwide, particularly in developing countries [1]. Risk factors for infectious gastroenteritis include exposure to various contaminated food products [2]. Several methods to detect pathogens directly in food samples have been reported [3, 4]; however, most food analysis requires a 25 g food sample. Ideally, pathogen detection in food should be at the single-cell level [5].

Several methods based on polymerase chain reaction (PCR) have been developed to detect a single-cell pathogen from enrichment culture [6–8]. Cocktail PCR, carried out in a single PCR tube for simultaneous detection of more than one bacterial target, has been investigated as a more cost-effective and time-saving method [9, 10]. However, it is difficult for small food laboratories to use ethidium bromide-based agarose gel. On the other hand, real-time PCR assays employing various types of fluorescence systems allow multiple detection during PCR [11–13]. This is an excellent method, but it requires an expensive real-time thermal cycler and reagents. Thus, small laboratories cannot afford this real-time method.

Another aspect of food analysis is the analysis time. Fresh food products must arrive to the market quickly, but current culture-based protocols require several days to confirm that the products are pathogen-free. Confirmation that fresh food is safe before shipping is, therefore, desired, but difficult in practice.

To solve these problems, we developed a quick cocktail PCR and dipstick DNA chromatography to differentiate PCR amplicons for *Salmonella enterica*, *Shigella* spp., enteroinvasive *Escherichia coli *(EIEC), and enterohemorrhagic *E. coli* (EHEC) from a single enrichment culture broth. In our previous report, we described a food pathogen enrichment (FPE) broth that supports the growth of *Campylobacter* without adding lysed blood and carbon dioxide [14]. The method detected a few *Campylobacter* cells in 25 g of chicken within 24 hours and was better than the conventional Bolton-based enrichment culture.

In this report, we describe a new protocol to detect *S. enterica*, *Shigella* spp., EIEC, and EHEC from the FPE broth. DNA preparation from the FPE broth was simplified and the cocktail PCR was designed to finish within 30 min. The PCR amplicon was visually differentiated using dipstick DNA chromatography within 5–10 min.

## 2. Materials and Methods

### 2.1. Bacterial Strains

The bacterial strains of *S. enterica*, *Shigella* spp., EIEC, and EHEC and other strains are listed in Table 1. All strains were supplied from the Gifu Type Culture Collection of the Microbial Genetic Resource Stock Center, Gifu University Graduate School of Medicine (Gifu, Japan), supported by the Ministry of Education, Culture, Sports, Science, and Technology of Japan. All strains were cultured on heart infusion agars (BD, Tokyo, Japan) at 37°C under an aerobic atmosphere overnight. A fresh culture was used for each experiment.

### 2.2. Determination of Optimal Enrichment Culture with FPE Broth

**Figure 1:**
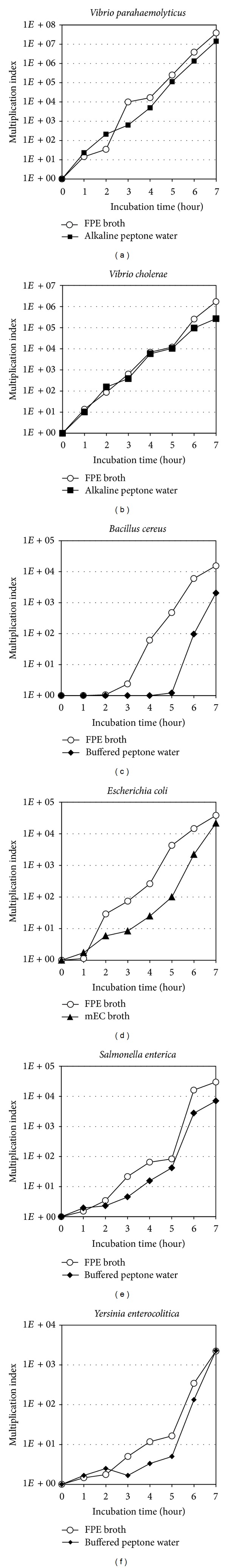
Growth of reference strains in FPE broth and established reference media. The initial number of bacteria was defined as one and the multiplication number is indicated on the *y*-axis. The *x*-axis represents incubation time.

FPE broth is designed to support the growth of *Campylobacter* species without blood and carbon dioxide [14]. This broth was used in the present study to simplify the total food analysis protocol because the FPE broth supported most food borne pathogens in our preliminary experiment. The growth of the foodborne pathogen in the FPE broth was compared with that in conventional selective enrichment broth (Figure 1). In the spiked ground meat experiment, diluted fresh bacterial solution and 25 g of beef were mixed in 225 mL of FPE broth and incubated at 37°C.

### 2.3. DNA Extraction

DNA was extracted from 1 mL of culture broth using a physical disruption method (MORA-EXTRACT, AMR, Gifu, Japan) according to the manufacturer's instructions, with a final DNA elution volume of 200–400 *μ*L.

DNA extraction from FPE broth was performed using a simplified protocol. One milliliter of 2 to 18 hours enrichment culture was collected in a 2 mL Eppendorf tube and centrifuged at 12,000 g. The supernatant was completely removed and 1 mL of T10E1 buffer was added and centrifuged under the same conditions. After the complete removal of the supernatant, 200 *μ*L of T10E1 buffer was added to the tube and mixed. The solution was transferred to a tube containing beads and physically disrupted for 1 min with a Disrupter Genie (Scientific Industry Inc., Bohemia, NY, USA). The tube was boiled at 100°C for 3 min. Five microliters of the solution were used for the cocktail PCR assay.

### 2.4. Cocktail PCR

The cocktail PCR conditions used in the present study are described below. The primers used are described in Table 2. PCR amplification was performed in 10 *μ*L of reaction mixture containing 5 *μ*L of 2× premix Ex *Taq* (Takara Bio, Shiga, Japan), 2.5 *μ*L of primer mixture, 0.5 *μ*L of distilled water, and 2 *μ*L of DNA template. PCR was carried out using the QuickBath thermal cycler (ThermoGen Ltd., Nagano, Japan) under the following conditions: 95°C for 3 min, 40 cycles of 95°C for 10 s, and 65°C for 10 s. The PCR cycles finished within 30 min.

### 2.5. Dipstick DNA Chromatography

**Figure 2:**
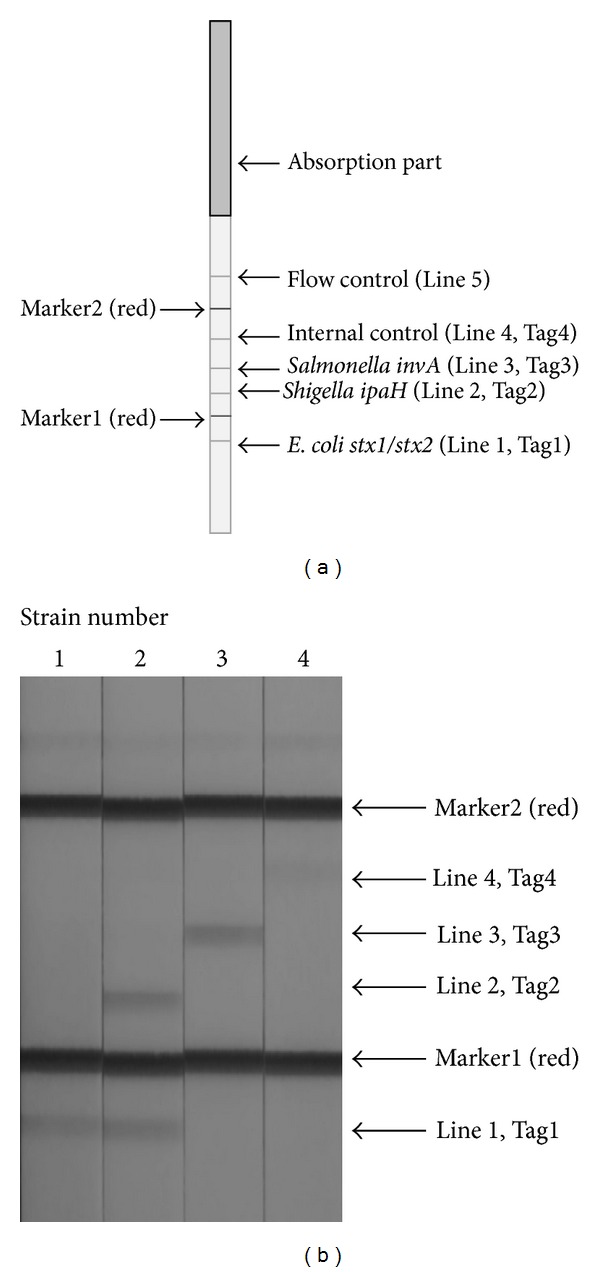
DNA strip for chromatography (a) and reaction results (b) for three pathogens and a negative control. (a) Line 1 (Tag1): EHEC, Line 2 (Tag2): *Shigella* spp. and EIEC, Line 3 (Tag3): *Salmonella* spp., Line 4 (Tag4): internal control, Line 5: flow control. (b) Strains: no. 1 *Eschesrichia coli* O157 (shiga toxin 1 and 2) GTC14510; no. 2 *Shigella dysenteriae* O1 (shiga toxin 1 and IpaH) GTC01057; no. 3 *Salmonella enterica* serovar Enteritidis GTC03838; and no. 4 internal positive control. All of amplicons are reacted with each tag.

The 5 terminus of the cocktail PCR amplicon was labeled with biotin, and the 3 terminus was labeled with four different tags. Streptavidin-coated blue latex, kindly provided from Fujikura (Saitama, Japan), bound the biotinylated 5 terminal amplicon and the tagged 3 terminus was bound on the antitag lines printed on the DNA strip (Figure 2).

After PCR, 30 *μ*L of streptavidin-coated blue latex solution was added to the PCR tube, and then the DNA strip was inserted into the PCR tube. The hybridized PCR amplicon was visualized in 5 to 10 min as a blue line, which represented the bound streptavidin-coated blue latex and biotin-labeled 5 terminus of the PCR amplicon.

### 2.6. A Protocol to Detect Multiple Foodborne Pathogens after 6 Hours Enrichment Culture of Ground Meat

For detection of foodborne pathogens with our protocol, 25 g of beef and 225 mL of FPE broth were homogenized in Stomacher bags (Eiken Chemical Co., Tokyo, Japan). The entire homogenate was transferred to a culture bottle and incubated with shaking at 37°C. After incubation, 1 mL of the supernatant was collected, and DNA was extracted using the physical disruption method described above. Subsequently, 5 *μ*L of the extracted DNA was analyzed by cocktail PCR primers (Table 2) using premix Ex *Taq* (Takara Bio, Shiga, Japan) and the QuickBath thermal cycler. After 30 min, the PCR amplicon was analyzed by dipstick DNA chromatography. Thirty microliters of streptavidin-coated blue latex solution were added to each tube. Subsequently, the DNA strip was inserted into each tube. After 5–10 min at room temperature, the amplicon bound on the appropriate line on the dipstick surface (Figure 2) was visualized by the blue latex of the biotin-labeled 5 terminus of the PCR amplicon.

### 2.7. Sensitivity and Specificity of the Cocktail PCR Dipstick DNA Chromatography (CPDC) Assay

To measure the sensitivity of the CPDC assay, purified chromosomal DNA of *E. coli *O157 GTC14510 and *S. enterica* serovar Enteritidis GTC03838 were prepared at six different concentrations (2 ng, 200 pg, 20 pg, 2 pg, 200 fg, and 20 fg) and assayed (Table 3). Another sensitivity assay starting from quantitatively diluted culture supernatants was also performed (Table 4). The specificity of the CPDC assay was determined using the 176 strains listed in Table 5.

### 2.8. Evaluation of CPDC Assay with Spiked Ground Meat

The CPDC assay after enrichment culture in FPE broth was compared with a commercial immunochromatography system (Wako Pure Chem. Industries, Ltd., Osaka, Japan). Ground beef collected from local supermarkets was immediately transported to our laboratory in an insulated cooler box at 4°C. However, the isolation frequency of the target *Shigella* spp., *Salmonella enterica*, and *E. coli *O157 : H7 was less than 0.1% by culture-based conventional methods in our preliminary experiments. We decided, therefore, to evaluate the CPDC assay with spiked ground meat experiments. Ground meat was collected from a supermarket and confirmed target to be pathogen free by conventional methods. One milliliter of a mixed culture containing *Shigella dysenteriae*, *S. enterica *subspecies *enterica *serovar Enteritidis, and *E. coli* O157:H7 at three different concentrations were mixed with 25 g of ground meat, and 225 mL of FPE broth was then added. The total volume was incubated at 37°C. Immediately, and 4 hours, 6 hours, 8 hours, and 18 hours, 1 mL of the enrichment was used for extraction and then used in the CPDC assay. Another aliquot was used for the commercial immunochromatography kit for *S. enterica* serovar Enteritidis and *E. coli* O157.

## 3. Results and Discussion

Conventional culture has been the “gold standard” method for the detection of enteric bacterial pathogens. The advantages of this method include identification, facilitation of outbreak management, and generation of an antimicrobial susceptibility profile [15]. However, this conventional method has many disadvantages. Many different enrichment broths and solid media are used to screen for all possible foodborne pathogens, and time-consuming protocols are prepared to generate a result. FPE broth is designed to support *Campylobacter* without adding lysed blood and carbon dioxide. *Campylobacter*, however, is a slow-growing organism and needs 24 hours to reach 10^4^ CFU/mL. Therefore, addition of selective antibiotics to the FPE broth was essential to suppress contaminating other bacteria for 24 hours enrichment culture. FPE broth could also support the growth of *Listeria* without adding blood, but the growth of *Listeria* is also slow, needing 24 hours to reach 10^4^ CFU/mL (unpublished data).

The growth of pathogens in conventional enrichment culture and FPE broths were measured (Figure 1). Approximately 1–10 bacteria were spiked in 225 mL of enrichment broth. *S. enterica *and *E. coli* reached 10^4^ CFU/mL after 6-hours incubation in FPE broth, buffered peptone water broth, and mEC broth, as shown in Figure 1. *V. parahaemolyticus* reached 10^4^ CFU/mL after 4-hours incubation in both FPE and alkaline peptone broths. Based on these data, we selected 6 hours incubation for the CPDC assay.

In the present study, cocktail PCR was capable of simultaneously determining the presence of *Salmonella* spp., *Shigella* spp., EIEC, and EHEC by targeting *invA*, *ipaH*, *stx1*, and *stx2* genes (Figure 2).

To evaluate the detection limit of the CPDC assay for each pathogen, 2 ng to 20 fg of DNA per reaction was prepared. The sensitivity and specificity of this assay are shown in Tables 3–5. The detection limit was 200 fg for each pathogen per CPDC assay (Table 3). The presence of the products with expected sizes was also confirmed by agarose gel electrophoresis, and nonspecific products were not observed (data not shown). The specificity of this CPDC assay was evaluated using 157 target strains (45 strains of *Shigella *spp., 54 strains of EHEC, 17 strains of EIEC, and 41 strains of *Salmonella *spp.) and 19 nontarget strains shown in Table 5. The detection limit of *Salmonella *and *Escherichia* from FPE culture supernatant (Table 4) was 10^3^ CFU/mL, while the commercial immunochromatography kit required 10^5^ CFU/mL. No false positive lines appeared on the dipstick DNA chromatography for any of the nontarget strains.

Immunochromatography is a simple technology to detect antigen in culture supernatant. However, immunochromatography targeting EHEC serotypes is not useful for testing food, because many kinds of *E. coli *serotypes produce shiga toxins. Thus, it is practically difficult to cover all of the EHEC serotypes by immunochromatography. Our method targeted both shiga toxin 1 and shiga toxin 2 toxins and detected non-O157 shiga toxin-producing serotypes (O26, O111, and O121). Three serotype (O45, O103, and O145) strains were not used because they are not available from our collection.

Shiga toxin 1 and shiga toxin 2 PCR products were designed to be bound on line 1 of the dipstick (Figure 2). The two genes were not equally amplified. The signal of the shiga toxin 2 amplicon was always bigger than the signal of the shiga toxin 1 amplicon.

The invasion plasmid antigen H (*ipaH*) gene is often used to diagnosis dysentery [16], because *ipaH* is carried by all four *Shigella* species as well as EIEC. In our cocktail primer, therefore, we selected the *ipaH*-specific primer to detect both *Shigella* and EIEC. The *ipaH* amplicon was designed to react on the second line of the dipstick chromatography strip, as shown in Figure 2. The CPDC assay was found to be effective at detecting *Shigella* species and EIEC from 4 to 6 hours FPE broth.

The CPDC assay required 4 to 6 hours FPE broth for the detection of *S. enterica* serovar Enteritidis. The detection limit of chromatography for *Salmonella *was, however, 10^5^ CFU/mL in culture supernatant. To reach this cell number in FPE broth, incubation for more than 6 hours was necessary (Table 6). There is another disadvantage to using immunochromatography. The commercially available immunochromatography kits for* Salmonella* serovars are limited. The products only detect serovar Enteritidis. Thus, it is difficult to screen many different *Salmonella* serotypes simultaneously, such as serovar Typhimurium, serovar Choleraesuis, serovar Dublin, serovar Typhi, and others.

The CPDC assay for *Salmonella* was evaluated by spiked ground meat experiments with three different inoculation levels from 2.5 to 10 CFU/25 g (Table 6). The CPDC assay detected target pathogens on the third line of a dipstick DNA strip from 4 to 6 hours culture with FPE broth. On the other hand, the commercial immunochromatography kit only detected antigens from 18 hours enrichment culture because the method requires 10^5^ CFU/mL of organism (Tables 4 and 6).

Multiplex PCR to detect many* Salmonella* serovars has been reported [17, 18]; however, we selected the *invA* gene for our assay because all *Salmonella* serovars carry this gene [19].

Internal amplification control (IAC) is designed to bind to line 4 of the dipstick chromatography strip to check for the presence of PCR inhibitor and false negatives [20]. A general guideline proposed for PCR testing of foodborne pathogens also requires the presence of IAC in the reaction mixture [21].

Systematic review of clinical implications, public health considerations, and the cost effectiveness of rapid diagnostic tests for detection and identification of bacterial pathogens in feces and food suggests that adoption of rapid test methods, especially for PCR, in combination with a routine culture is unlikely to be cost-effective [7, 22]. However, as the cost of rapid technologies decreases, total replacement with rapid technologies may be feasible.

The clinical impact of the decreased turnaround time means that bacterial diarrhea is more promptly ruled out using the CPDC assay compared to using conventional culture in small laboratories. This reduces the expenditure of infection control resources and, in particular, in cases of sporadic diarrhea, helps to reduce the requirement for scarce isolation rooms. In addition, the earlier availability of results is helpful in community-based management of outbreaks.

For detection of *Salmonella* spp., *Shigella* spp., EIEC, and EHEC, the overall time to confirm a positive result by conventional culture plus immunochromatography is at least two working days. Generating a negative report requires 48 hours. In contrast, a report can be generated for the CPDC assay within one working day. One advantage of an early laboratory report is early judgment of contamination, which can prevent food poisoning and additional outbreaks. The method also contributes to the quick shipment of fresh food products to the markets.

## 4. Conclusion 

Cocktail PCR targeting multiple foodborne pathogens and simple dipstick DNA chromatography to differentiate the PCR product was designed. The method was applied to detect pathogens in ground meat after 6 hours enrichment broth, which supports the growth of broad range foodborne pathogens. This single tube PCR and enrichment method help to simplify food analysis protocol. As a result, the method offers rapid report to food suppliers and helps the quick shipment of safety-confirmed food products to markets.

## Figures and Tables

**Table 1:** List of bacterial strains used in this study.

Bacteria	Serotype	Toxin	Strains	Number of strain
EHEC (Enterohemorrhagic *E*. *coli*)	O26: H-	Shiga toxin 1	GTC14538, GTC14548, GTC14605, GTC14606	4
	O26: H11	Shiga toxin 1	GTC14516, GTC14540, GTC14549, GTC14557, GTC14558	5
		Shiga toxin 1 and 2	GTC14515, GTC14539, GTC14567	3
	O111: H-	Shiga toxin 1	GTC14517, GTC14528, GTC14541	3
		Shiga toxin 1 and 2	GTC14508, GTC14582	2
	O115: H10	Shiga toxin 1	GTC14518	1
	O119: H2	Shiga toxin 1	GTC14529	1
	O121: H19	Shiga toxin 2	GTC14530, GTC14577, GTC14601, GTC14602	4
	O128: H-	Shiga toxin 1 and 2	GTC14603	1
	O157: H7	Shiga toxin 2	GTC14513, GTC14514,GTC14524, GTC14525, GTC14537 GTC14546, GTC14547, GTC14550, GTC14553, GTC14560	10
		Shiga toxin 1 and 2	GTC14510, GTC14511, GTC14512, GTC14521, GTC14535 GTC14536, GTC14544, GTC14545, GTC14551, GTC14552,	10
	O157: H-	Shiga toxin 1 and 2	GTC14507, GTC14520, GTC14530, GTC14543, GTC14555 GTC14556, GTC14566, GTC14571, GTC14587, GTC14588	10
EIEC (Enteroinvasive *E*. *coli) *	O28: H-		GTC14240, GTC14243, GTC14251, GTC14259, GTC14260	5
	O124: H-		GTC14241, GTC14242, GTC14245, GTC14262	4
	O136: H-		GTC13248, GTC14254	2
	O144: H-		GTC14249, GTC14252, GTC14256	3
	O164:H-		GTC14244, GTC14246, GTC14247,	3
*Salmonella enterica *				
subsp.*enterica*	serovar Typhimurium		GTC02557, GTC02561, GTC02562, GTC02563, GTC02564	10
			GTC02571, GTC02572, GTC-2573, GTC02574, GTC02575	
subsp.*enterica *	serovar Enteritidis		GTC03838, GTC00131, GTC02377, GTC02382, GTC02387	7
			GTC02389, GTC02390	
subsp.*enterica *	serovar Dublin		GTC13214, GTC13215, GTC13216, GTC13217,GTC13218	10
			GTC13219, GTC13220, GTC13221. GTC02558, GTC02560	
subsp.*enterica *	serovar Typhi		GTC3P001, GTC3P074,GTC3P076, GTC3P081, GTC3P085	10
			GTC3P087, GTC3P091, GTC3P095, GTC3P100, GTC3P106	
subsp.*enterica *	serovar ParaTyphi A		GTC3P002, GTC3P082, GTC3P083	3
*Salmonella bongori *			GTC03793T	1
*Shigella boydii *			GTC00779T, GTC01912, GTC01913, GTC01914, GTC01915	8
			GTC01915, GTC01916, GTC01917,	
*Shigella dysenteriae *			GTC01057T, GTC00786, GTC01929, GTC01930, GTC14808	17
			GTC14809, GTC14810, GTC14811, GTC14812, GTC14813,	
			GTC14814, GTC14815, GTC14816, GTC14817, GTC14818,	
			GTC14819, GTC14820,	
*Shigella flexneri *			GTC 0780T, GTC01918, GTC01920, GTC02007, GTC02008	13
			GTC02009, GTC02010, GTC02011, GTC02012, GTC02013,	
			GTC02015, GTC02016, GTC02014,	
*Shigella sonnei *			GTC00781T, GTC01909, GTC01910, GTC01911, GTC01931	7
			GTC01932, GTC01933,	
*Escherichia coli *			GTC00503 T	1
*Escherichia albertii *			GTC 16441T	1
*Escherichia fergusonii *			GTC 01720T	1
*Escherichia vulneris *			GTC 10613T	1
*Escherichia hermannii *			GTC 10612T	1
*Escherichia blattae *			GTC 01342T	1
*Citrobacter freundii *			GTC 14890T	1
*Citrobacter diversus *			GTC 00114T	1
*Citrobacter rodentium *			GTC 14911T	1
*Citrobacter youngae *			GTC 14914T	1
*Klebsiella pneumoniae *			GTC 14868T	1
*Enterobacter cloacae *			GTC 00109T	1
*Enterobacter aerogenes *			GTC 14962T	1
*Cronobacter sakazakii *			GTC 14952T	1
*Serratia marcescens *			GTC 14672	1
*Yersinia enterocolitica *			GTC 00127T	1
*Pseudomonas aeruginosa *			GTC 00002T	1
*Vibrio parahaemolyiticus *			GTC 02055	1
*Staphylococcus aureus *			GTC 00286T	1

GTC is the Gifu Type Culture Collection supported by the National Bioresource Project (NBRP: http://www.nbrp.jp/) of the Ministry of Education, Culture, Sports, Science,and Technology.

“T” after the strain number means type strain.

**Table 2:** Cocktail primer list.

Pathogen	Targeted gene	Primer name	Sequence (5′-3′)
EHEC	*stx1 *	Forward *stx1 *	Biotin-ACAGGATTTGTTAACAGGAC
		Reverse *stx1 *	Tag1-TCTGTATTTGCCGAAAACGT
	*stx2 *	Forward *stx*2	Biotin-GATACAGAGAGAATTTCGTC
		Reverse *stx*2	Tag1-GCCAGTTATCTGACATTCTG
*Shigella *spp. and EIEC	*ipaH *	Forward *ipaHF *	Biotin-CTCGCAGAGAAACTTCAGCTCT
		Reverse *ipaHR *	Tag2-TTCTCTTCACGGCTTCTGACCAT
*Salmonella *spp.	*invA *	Forward *invA *	Biotin-TGACAGAATCCTCAGTTTTTCA
		Reverse *invA *	Tag3-AGATAAGACGGCTGGTACTGAT
Internal control		Forward IPC	Biotin-ACTCTTCCTAGCAGGATCCCTCTAAG
		Reverse IPC	Tag4-GCAATTCTAATACGACTCACTATAGG

**Table 3:** Detection sensitivity of CPDC assay.

DNA concentration	EHEC serovar O111, GTC14517 (*stx*1)	EHEC serovar O157, GTC14513 (*stx*2)	*Shigella dysenteriae *serovar 2, GTC01929 (*ipaH*)	*Salmonella enterica* serovar Enteritidis GTC03838 (*invA*)
2 ng/assay	+	+	+	+
200 pg/assay	+	+	+	+
20 pg/assay	+	+	+	+
2 pg/assay	+	+	+	+
200 fg/assay	+	+	+	+
20 fg/assay	−	−	−	−

Serially diluted purified DNA of each strain was used to count detection sensitivity.

**Table 4:** Detection sensitivity of CPDC assay and immunochromatography.

Bacterial concentration (CFU/mL)	EHEC O157 GTC14510		Bacterial concentration (CFU/mL)	*Salmonella enterica* serovar Enteritidis GTC03838	
	Immunochromat.	CPDC assay		Immunochromat.	CPDC assay
3.3 × 10^9^	+	+	2.5 × 10^9^	+	+
3.3 × 10^8^	+	+	2.5 × 10^8^	+	+
3.3 × 10^7^	+	+	2.5 × 10^7^	+	+
3.3 × 10^6^	+	+	2.5 × 10^6^	+	+
3.3 × 10^5^	+	+	2.5 × 10^5^	+	+
3.3 × 10^4^	−	+	2.5 × 10^4^	−	+
3.3 × 10^3^	−	+	2.5 × 10^3^	−	+
3.3 × 10^2^	−	−	2.5 × 10^2^	−	−
3.3 × 10	−	−	2.5 × 10	−	−
3.3	−	−	2.5	−	−

**Table 5:** Specificity of CPDC assay.

Bacteria	Serotype (Virulence factor)	Reacted Dipstick line	CPDC assay (positive/strains)
EHEC	O26: H- (*stx*1)	Line 1	4/4
	O26: H11(*stx*1)	Line 1	5/5
	O26: H11(*stx*1/2)	Line 1	3/3
	O111: H- (*stx*1)	Line 1	3/3
	O111: H- (*stx*1/2)	Line 1	2/2
	O115: H10(*stx*1)	Line 1	1/1
	O119: H2(*stx*1)	Line 1	1/1
	O121: H19(*stx*2)	Line 1	4/4
	O128: H- (*stx*1/2)	Line 1	1/1
	O157: H7(*stx*2)	Line 1	10/10
	O157: H7(*stx*1/2)	Line 1	10/10
	O157: H-(*stx*1/2)	Line 1	10/10
EIEC	O28: H- (*IpaH*)	Line 2	5/5
	O124: H- (*IpaH*)	Line 2	4/4
	O136: H- (*IpaH*)	Line 2	10/10
	O144: H- (*IpaH*)	Line 2	3/3
	O164:H- (*IpaH*)	Line 2	3/3
*Shigella boydii *	(*IpaH*)	Line 2	8/8
*Shigella dysenteriae *	(*IpaH*)	Line 2	16/16
*Shigella flexneri *	(*IpaH*)	Line 2	13/13
*Shigella sonnei *	(*IpaH*)	Line 2	7/7
*Salmonella enterica *			
subsp.*enterica *	serovar Typhimurium (*InvA*)	Line 3	10/10
subsp.*enterica *	serovar Typhi (*InvA*)	Line 3	10/10
subsp.*enterica *	serovar Enteritidis (*InvA*)	Line 3	7/7
subsp.*enterica *	serovar Dublin (*InvA*)	Line 3	10/10
subsp.*enterica *	serovar paratyphi A (*InvA*)	Line 3	3/3
*Salmonella bongori*GTC 03793 T	(*InvA*)	Line 3	1/1
*Escherichia coli *GTC00503 T		N*	0/1
*Escherichia albertii*GTC 16441 T		N	0/1
*Escherichia fergusonii*GTC 01720 T		N	0/1
*Escherichia vulneris* GTC 10613 T		N	0/1
*Escherichia hermannii*GTC 10612 T		N	0/1
*Escherichia blattae*GTC 01342 T		N	0/1
*Citrobacter freundii*GTC 14890 T		N	0/1
*Citrobacter diversus*GTC 00114 T		N	0/1
*Citrobacter rodentium *GTC 14911 T		N	0/1
*Citrobacter youngae*GTC 14914		N	0/1
*Klebsiella pneumonia *GTC 14868 T		N	0/1
*Enterobacter cloacae*GTC 00109 T		N	0/1
*Enterobacter aerogenes*GTC 14962 T		N	0/1
*Cronobacter sakazakii*GTC 14952 T		N	0/1
*Serratia marcescens*GTC 14672		N	0/1
*Yersinia enterocolitica*GTC 00127 T		N	0/1
*Vibrio parahaemolyticus *GTC02055		N	0/1
*Pseudomonas aeruginosa*GTC 00002 T		N	0/1
*Staphylococcus aureus*GTC 00286 T		N	0/1

N*: no positive line.

Reacted line no. 1 is *stx*1 and 2 for EHEC, line 2 is *ipaH* for* Shigella* spp. and EIEC, and line 3 is for *invA* for *Salmonella*.

**Table 6:** Result of CPDC assay in spiked ground meat samples.

Inoculated level (CFU/25 g)	CPDC assay					Immunochromatography				
	Enrichment time (h)					Enrichment time (h)				
	0	4	6	8	18	0	4	6	8	18
	Enterohemorrhagic *E. coli *O157 GTC14510									
13.3	−	−	+	+	+	−	−	−	−	+
6.7	−	−	+	+	+	−	−	−	−	+
3.3	−	−	+	+	+	−	−	−	−	+
Control	−	−	−	−	−	−	−	−	−	−

	*Salmonella enterica* serovar Enteritidis GTC03838									
10.0	−	+	+	+	+	−	−	−	−	+
5.0	−	+	+	+	+	−	−	−	−	+
2.5	−	+	+	+	+	−	−	−	−	+
Control	−	−	−	−	−	−	−	−	−	−
